# “It’s just another tool on my toolbelt”: New York state law enforcement officer experiences administering naloxone

**DOI:** 10.1186/s12954-023-00748-3

**Published:** 2023-03-06

**Authors:** Danielle Lloyd, Kirsten Rowe, Shu-Yin John Leung, Elham Pourtaher, Kitty Gelberg

**Affiliations:** 1 Office of Program Evaluation and Research, New York State Department of Health, AIDS Institute, Corning Tower, Room 342, Albany, NY 12237 USA; 2 Office of Drug User Health, New York State Department of Health, AIDS Institute, Corning Tower, Room 474, Albany, NY 12237 USA; 3 Vermont, USA

**Keywords:** Harm reduction, Law enforcement, Opioids, Naloxone, People who use drugs

## Abstract

**Background:**

Although naloxone is widely acknowledged as a life-saving intervention and a critical tool for first responders, there remains a need to explore how law enforcement officers have adapted to a shifting scope of work. Past research has focused mainly on officer training, their abilities to administer naloxone, and to a lesser extent on their experiences and interactions working with people who use drugs (PWUD).

**Methods:**

A qualitative approach was used to explore officer perspectives and behaviors surrounding responses to incidents of suspected opioid overdose. Between the months of March and September 2017, semi-structured interviews were conducted with 38 officers from 17 counties across New York state (NYS).

**Results:**

Analysis of in-depth interviews revealed that officers generally considered the additional responsibility of administering naloxone to have become “part of the job”. Many officers reported feeling as though they are expected to wear multiple hats, functioning as both law enforcement and medical personnel and at times juggling contradictory roles. Evolving views on drugs and drug use defined many interviews, as well as the recognition that a punitive approach to working with PWUD is not the solution, emphasizing the need for cohesive, community-wide support strategies. Notable differences in attitudes toward PWUD appeared to be influenced by an officer’s connection to someone who uses drugs and/or due to a background in emergency medical services.

**Conclusion:**

Law enforcement officers in NYS are emerging as an integral part of the continuum of care for PWUD. Our findings are capturing a time of transition as more traditional approaches to law enforcement appear to be shifting toward those prioritizing prevention and diversion. Widespread adoption of naloxone administration by law enforcement officers in NYS is a powerful example of the successful integration of a public health intervention into police work.

## Introduction

Evidence-based interventions, enhanced cross-sector partnerships, and shifts in institutional norms have been cornerstones of the opioid overdose response. While such interventions appeared to be having an impact on reducing deaths due to opioids [1], recent data has shown a rapid acceleration of drug overdose deaths, primarily due to illicitly manufactured fentanyl and possibly the complex effects of the SARS-CoV-2 pandemic [2, 3]. According to recent provisional data from the CDC, over 108,000 drug overdose deaths were reported in the 12 months ending in February 2022 [4]. This represents the highest number of overdose deaths ever recorded in a 12-month period.

Increasing access to naloxone is widely acknowledged as a lifesaving intervention and expanding awareness and availability must continue to play a vital role in response efforts [5]. A fast and effective antidote for opioid overdose with no potential for abuse, naloxone reduces respiratory depression within minutes of administration [6, 7]. The advent of the intranasal formulation resulted in increased accessibility to individuals without a medical background [8]. Recognized as a critical harm reduction tool, naloxone is just one of a multitude of evidence-based options available to mitigate risks associated with drug use.

As the opioid overdose crisis worsened in the late 2000s, it became evident that some of the most well-positioned individuals to respond quickly to suspected overdoses were law enforcement officers. This is particularly true in rural areas with limited emergency medical services (EMS) capacity. In 2010, the Office of National Drug Control Policy called for first responders to carry and administer naloxone, stating “naloxone should be in the patrol cars of every law enforcement officer across the nation” [9]. Since then, naloxone has increasingly been used by police officers, emergency medical technicians, and non-emergency first responders to reverse opioid overdoses [5]. The Bureau of Justice Assistance (BJA), in their “Law Enforcement Naloxone Toolkit,” highlighted the potential benefits of law enforcement naloxone programs to both officers and their agencies as well as the public at large. Beyond the obvious benefit of reducing overdose related mortality, the BJA noted “improved job satisfaction” among individual officers who were presented with the option to “do something” at the scene of an overdose [10]. Engaging law enforcement in these efforts was an acknowledgement that traditional approaches to policing, including the criminalization and prosecution of people who use drugs (PWUD), would not effectively curb rates of fatal overdose.

Peer-reviewed studies suggest law enforcement naloxone programs show promise, and that officers have been generally receptive to embracing such a harm reduction approach [8, 10–12]. These programs have the potential to strengthen community bonds with police [8], with many introducing officers to harm reduction training which may result in changing attitudes toward PWUD [13, 14]. Relatively recent evidence based-strategies such as the 911 Good Samaritan Law (GSL) allows people to call 911 during a drug or alcohol-induced overdose without fear of arrest [15] indicates a progressive shift toward harm reduction and drug decriminalization. Such initiatives, also referred to as drug immunity laws, have become increasingly common in the U.S. As of 2017, 40 states and the District of Columbia had enacted some form of this law with the scope of immunity varying by state [16].

In addition to the noted successes, various challenges with law enforcement-administered naloxone programs have also been explored in the literature. According to Winograd et al. [17], overdose education training resulted in mixed changes in attitudes toward overdose victims, resulting in positive post-training attitudes for the majority (55.3%), but negative or no changes in attitudes among the remaining 43.7% of participating officers. Additionally, some factors may inhibit program effectiveness including law enforcement officers’ negative feelings about the drug possession component of the GSL [18], bystanders’ fear of arrest despite their awareness of the GSL [19] and their concerns regarding potential post-naloxone agitation [20]. Although critically important in providing PWUD protection from arrest and preventing overdose deaths [21], there are notable shortcomings of drug immunity laws such as requirements that immunity be contingent upon cooperation with police or involvement in drug treatment [22]. The New York law, as it is currently written, does not afford individuals protection from a violation of probation or parole or if there are open warrants for arrest [15].

## Efforts to increase access to naloxone in New York state

In 2014, multiple New York state (NYS) agencies formed a public health/public safety partnership to increase statewide access to naloxone. The New York Police Department’s (NYPD) naloxone program operates under the umbrella of the New York City Department of Health and Mental Hygiene (NYCDOHMH), and therefore was not involved in this collaboration. The NYS Department of Health (NYSDOH), Division of Criminal Justice Services (DCJS), Albany Medical Center, the Harm Reduction Coalition, and the Office of Addiction Services and Supports (OASAS) collaborated to develop and deliver a program to train and equip law enforcement officers to administer naloxone in the event of a suspected opioid overdose.

Law enforcement officers from agencies participating in the program complete the NYS Public Safety Naloxone Quality Improvement Usage Report (hereafter, usage report) after each naloxone administration and submit it to NYSDOH. Between program inception in April 2014 and April 2021, DCJS provided naloxone administration training to over 12,000 officers from 631 law enforcement agencies across the state. During this period, 11,546 usage reports were submitted to the NYSDOH by officers from 647 agencies in 62 NYS counties [23]. Of those individuals aided by officers, 88% lived [23]. The program remains active and continues to collect and manage data on all reported naloxone administrations in the state.

Given their roles as first responders, it is necessary to explore how officers have been adapting to a shifting scope of work, in many cases counter to traditional approaches to policing. A growing body of research suggests that law enforcement officers are receptive to naloxone training, increasing knowledge, changing their attitudes over time, and improving overall drug governance [11, 24]. Although the benefits of, and barriers to, law enforcement administration of naloxone are well documented, there remains much to be learned about officer perspectives on these relatively new and critical roles and responsibilities, and their interactions with individuals at often highly complex overdose scenes.

## Methods

A qualitative methodology was adopted to examine individual officer perspectives and behaviors surrounding naloxone administration using semi-structured, in-person interviews. Topics addressed in the interview guide included training received, officer experiences working with PWUD and responding to scenes of suspected overdoses, post-naloxone administration protocols, and knowledge of applicable laws and regulations. Although the initial target was 30 interviews, the research team was able to exceed the target for a total of 38 interviews due to a robust recruitment strategy.

## Participant recruitment

Participants were recruited from law enforcement agencies throughout NYS, with the exception of the five boroughs of New York City which were excluded due to law enforcement naloxone training occurring under the jurisdiction of NYCDOHMH. A multi-pronged approach to participant recruitment was used, relying on existing connections to multiple law enforcement entities to ensure diverse perspectives were captured throughout rural and urban areas of the state. For example, the Executive Director of the NYS Association of Chiefs of Police emailed the member agencies requesting participation, and direct contact was made with law enforcement agencies registered with NYSDOH and officers who submitted usage reports. Recruitment took place on a rolling basis during the data collection period.

Eligibility criteria included employment by a NYS law enforcement agency, consisting of local law enforcement agencies, sheriff’s departments, state troopers or college campus police, prior completion of naloxone training and reported administration of at least one dose of naloxone to NYSDOH within the past 6 months. Efforts were made to recruit officers who reported multiple administrations.

### Ethics and informed consent

Prior to conducting interviews, the study protocol was reviewed and approved by the NYSDOH Institutional Review Board (IRB). Participants were not compensated for their time, as all had received permission from a supervising officer to participate in interviews during working hours. All participants were provided with an informed consent document and provided verbal consent before the interview commenced.

### Data collection

Participant interviews were scheduled through the law enforcement agency with which the officer was affiliated. When possible, interviews were conducted in person at the agency, most often in a conference room or a private office setting. All efforts were made to interview participants during scheduled shifts, including early mornings, evenings, and weekends. On a few occasions, participants switched shifts to accommodate the interviewer’s schedule, as multiple interviews needed to be coordinated in the same region on the same day.

The interview guide was developed by staff from the NYSDOH Office of Drug User Health and the Office of Program Evaluation and Research. At the beginning of the interview, participants were asked to complete a brief survey which helped guide the discussion. The survey included demographic questions and addressed the frequency of prior naloxone administrations.

Data collection took place between March and September 2017. All interviews were conducted by two evaluation staff from the NYSDOH who had been trained in the use of best practices for in-depth interviewing. Interviews were audio recorded, unless refused by the participant. The interviews took an average of 1 hour to complete.

### Data analysis and storage

The analysis conducted was exploratory in nature, with the intent of better understanding officer attitudes surrounding their roles and responsibilities in the administration of naloxone and associated interactions with PWUD. We took an inductive approach to our research, and the semi-structured format of our interview guide allowed for rich discussion, permitting us to gain insight into nuanced perspectives; perspectives which could not otherwise be captured through survey research.

Upon completion of each interview, interviewer debriefing sessions were held. During these sessions, the interviewers reviewed and compared notes taken during the interviews, highlighted points of interest and, as interviews progressed, identified potential themes emerging from the data. These debriefing sessions and interviewer notes were critical in contributing to the analysis, as points of interest were identified and catalogued, forming the foundation for subsequent review of the transcripts. These sessions and notes were particularly valuable in instances where participants declined the audio recording of an interview.

Anonymized audio recordings of interviews were submitted on a rolling basis to a third-party transcription service for verbatim transcription. Once the transcripts were received, they were reviewed for accuracy and imported into NVivo 10 for analysis. We utilized a Grounded Theory approach for categorizing and comparing qualitative data [25, 26]. This iterative method allows for simultaneous coding and analyzing, as well as the continuous sorting of the data [26]. Codes were identified and categorized into themes as transcript data was examined. Multiple stages of collecting, refining and categorizing the data were carried out, allowing for comparisons between the existing and emerging themes.

All data were stored and managed by the NYSDOH Office of Program Evaluation and Research. All participants were assigned unique identifiers, and no identifying information was linked to any audio recordings, transcripts or interviewer notes.

## Findings

**Fig. 1:**
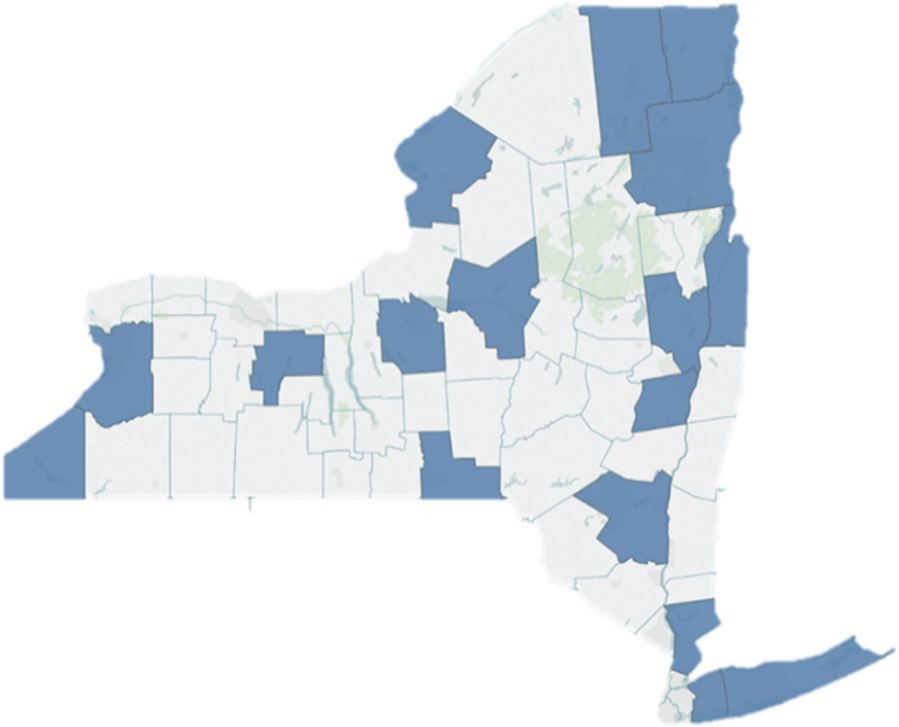
Map of New York state depicting counties where participating law enforcement agencies are located. Counties in this study include Albany, Broome, Chautauqua, Clinton, Erie, Essex, Franklin, Jefferson, Nassau, Oneida, Onondaga, Ontario, Saratoga, Suffolk, Ulster, Washington and Westchester

A total of 38 semi structured interviews were conducted across 17 counties at 23 law enforcement agencies (Fig. 1). Of these interviews, 34 took place in person and four were conducted via phone. Thirty-three participants gave permission for audio recording, while the remaining five participants requested that the interviewers take notes only. In these instances, notes were used during the analysis in place of a transcript. Participating agencies ranged in size from 12 to 625 staff, with a median size of 39. Most participants identified themselves as patrol officers, although a few were in supervisory positions such as a sergeant or a lieutenant. The majority of participants were male, with an average of 10 years of service as a law enforcement officer. Based upon participant estimates, the mean number of naloxone administrations among participants over the span of their careers was 8.6. Nearly 90% of participants interviewed were trained to carry and administer naloxone in 2014 or thereafter, coinciding with the first year of the New York state public safety naloxone training curriculum. Four participants were trained in naloxone administration before 2014 and reported receiving prior overdose training as emergency medical technicians or paramedics (Table 1).

**Table 1:** Participant characteristics (*n* = 38)

Variable	Value
*Sex*	
Male	34
Female	4
*Job title*	
Patrol officer	27
Sergeant	2
Lieutenant	2
Chief	1
Sheriff’s deputy	6
*Years in law enforcement*	
0–5	12
6–10	11
11–15	6
16–20	4
21+	2
UK	3
*Year trained to administer naloxone*	
Prior to 2014	4
2014 and onward	32
UK	2
*Total naloxone administrations during career*	
Less than 5	20
5–9	7
10 or more	11

### Perceived roles and responsibilities associated with naloxone administration

#### Naloxone administration is becoming “part of the job”

When participants were asked about their roles and responsibilities in responding to suspected overdoses, there was a general understanding that the additional responsibility of administering naloxone is yet another lifesaving action that had recently become “part of the job”. This perspective seemed to allow participants to examine the act of administration, given that it was largely considered to be within their scope of work.

“We’re first responders. It is what it is. You don’t get to pick what you like or don’t like to do. I mean, I do it [administer naloxone]. I show up. ‘Cause that’s what I have to do.” JC-06-01

Although no officer interviewed reported refusing to carry naloxone at any point, some participants reported being aware of other officers who would rather not administer naloxone if given a choice. In response to these attitudes at some agencies, naloxone is primarily administered by a select officer on a given shift referred to as the “Narcan Man” by some colleagues. This officer appears to be willing to shoulder the burden of repeat naloxone administrations, due to either a sense of personal or professional responsibility.

…but [what is our job?] to serve and protect. And I wonder if officers who have a problem administering Narcan also have a problem carrying this AED.” CD-01-01

“When I first heard about it [the need to administer naloxone], yeah, I was the cynic and I didn’t like that. But the more I’ve seen it, you know, it’s the bigger picture. Who cares about that arrest at the end of the day?” – CD-07-03

#### Non-traditional approaches to policing

Many participants reported feeling as though they are expected to wear multiple hats, functioning as both law enforcement officers and medical personnel. Some described what they considered the “social work” component of their work, which may have been amplified by the opioid crisis.

“21st century policing is very unique…you’re doing, from one call [to the next], you can be family counselor and then next you have to be a fireman, then next you have to be searching someone’s house for a burglar or something like that.” CD-01-01

“…and like I said, I have the understanding and sentiment of it [responding to an overdose], but you can’t ask someone to enforce the law, you know, fairly…and then ask them to do a function of social work.”-BC-06-02

#### Shifting expectations of officers

Such scenarios posed challenges for some participants, as they sometimes felt they juggled contradictory roles. Additional challenges were presented when the individual who was revived after an overdose or the bystanders on the scene prove unwilling to provide any additional information about the source of the drug. This, at times, further complicates the participant’s relationships with PWUD. Many participants reported feeling compelled to unearth the source of the “bad batch” to prevent others from overdosing and work toward the eventual identification and arrest of the dealer, rather than focusing efforts on arresting the individual who was using.

“You are pretty much being told: now you’re going in as a dual person. It’s not everybody…but they [other officers] don’t know how to deal with these new directives.”—WA-01-01

The enactment of the 911 Good Samaritan Law (GSL) and the directive to carry naloxone has resulted in some officers feeling as though their priorities are conflicting. Some participants felt the law should be changed to include some repercussions for possession, with the idea that it would lead to some level of behavior change.

“I think, getting rid of, or at least revamping, the Good Samaritan Law, will help…the judge we have, a lot of times…part of their sentence will be that they are mandated to drug court.”—CD-07-01

While all participants reported being familiar with the GSL and its protections for PWUD, some reported an unclear understanding of the law in its entirety, citing “grey areas”. These misunderstandings seemed to be common, leaving room for interpretation, particularly when it came to the amount of a substance that constituted personal use and whether the GSL could protect individuals on parole.

“He overdosed. He was on parole. So, I haven’t had any contact with him since because his parole officer violated him, and he went back to prison.”—PB-01-01

### Experiences at the scene of a suspected overdose

#### The administration process

Participants’ initial experiences administering naloxone were often reported as the most challenging, usually due to the stressors associated with a life-or-death situation and lack of familiarity with assembling the two-piece nasal administration device, which required attaching a vial to the atomizer. Although this kit has been superseded by the one-piece kit that does not require assembly at the scene, many participants who were trained to administer naloxone early on in the program learned to use the two-piece device. Performing in such circumstances, often at a chaotic scene, resulted in some initial difficulties with kit assembly. However, this was most commonly reported about administrations soon after training, with participants becoming more comfortable with the assembly and administration processes on subsequent calls.

“To be honest, there’s so much stuff going on…I have family members screaming. And they’re pissed off at the guy on the floor. And then “Save him.” And you’re dealing with that guy at the same time the radio is squawking and then EMS is finally rolling in or fire is showing up…[the most difficult thing] is about keeping track of how long it was from the time you gave him the first dose to the time I’m supposed to give him the second dose.”—CD-07-03

#### A team response

Participants acknowledged some level of difficulty dealing with the same individual who had overdosed multiple times; however, these experiences did not impact willingness to respond to calls for assistance. Although most participants did not report personally administering naloxone to the same person on multiple occasions, many participants reported encountering the same person overdosing on different occasions as part of an overdose response “team”.

These team responses were often described as multiple officers, at times from different agencies, joined by EMS or the local fire department. Other responders will play supporting roles to secure the scene, manage bystanders, and assist with naloxone device assembly and administration. At larger departments, many calls are answered by a pair of officers, or an officer will arrive to provide back up support soon after the initial call is answered. In some jurisdictions, it is common for agencies from neighboring communities to also respond and ensure that no additional assistance is needed.

“And a lot of times more [first responders] will show up. So the chance of somebody being alone [at the scene of an OD] is extremely rare.”—CD-07-01.

#### Bystander involvement

Unpredictable and challenging response scenes were often referenced by participants, ranging from small spaces in which the individual who overdosed needed to be moved before administration, to raucous scenes with many bystanders. The need to secure the scene and ensure the safety of all present prior to the administration of naloxone was a constant concern, and often cited by participants as a priority. Participants shared examples of firearms at the scene, multiple individuals who had overdosed, and at times, needing to tend to children who were present.

“…it’s never a good feeling as an officer, because I’ve been in that situation where I’ve been trying to arrest someone and then [someone puts their hands on you]. So…I don’t like that feeling knowing people are behind me that I don’t have my eyes on.”—PB-01-01

Participants typically described encounters with bystanders at the scene of an overdose as routine but occasionally difficult to manage. The latter was particularly true when concerned friends or family were present.

“It’s sad, because, especially when a person has found them, that’s the last thing they want to see. You’re kinda like “Aw man, I wish that’s not how you saw them. I’d say the more difficult ones are the ones that the family didn’t know.”—CC-23-01

Some participants shared frustrations about bystanders who refused to divulge information about the quantity or type of drug used. Frequently, individuals would leave the scene of the overdose before law enforcement arrived, perhaps due to fear of interrogation or arrest despite protections afforded by the GSL. Some participants expressed preoccupation about their activities being recorded by bystanders.

“No [bystander] has really affected us, besides filming and getting too close with the camera when we are trying to work…it’s very, very intrusive on that particular patient when they’re unconscious and someone is filming and not helping.”—YK-01-01.

### Factors that shape officer attitudes

Evolving views on drugs and drug use defined many of the interviews. It was widely recognized that a punitive approach to working with PWUD is not the solution; participants mostly recognized that they could not “arrest their way out of this” epidemic, and many emphasized the need for cohesive, community-wide support strategies.

#### Medical background

Those with EMS or other medical training tended to describe addiction as a disease rather than a moral failing when recalling encounters with PWUD, and generally displayed more confidence in responding to a suspected overdose scene.

“You're not there for a crime or anything like that. You're there to actually medically help them out. But, the next day they'll send you there again. So, it's a little tough on guys too. I guess you could say in that regard, especially for the person. That's who you really feel bad for.”—YK-01-01

“If you can reduce the transmission of Hepatitis C, I mean, I'm all for it. I mean the reality of it is—I mean it's just a clean needle. I mean what do we—we're not potentiating the problem, we're helping stop public health problems.”—BC-06-02

#### Personal connections

There were some notable differences in attitudes toward PWUD, which appear to be influenced by a participant’s connection to someone who uses drugs. These experiences often appeared to result in increased empathy for those who have overdosed.

Participants with personal connections to friends or family members who have used drugs tended to describe their encounters with people who have overdosed in compassionate, human-centered terms:

“You know, I had people I know through the community that have overdosed and—or died, and family member that have had those issues. So the human side of me—you know, I want to try to help them.”—CM-07-01

“In a way [administering naloxone so many times] had changed me. It’s really tough. I have people that are close to me, a couple family members that are very talented, young, beautiful women—that have gotten into it.”—CD-07-02

Those who reported personal connections to PWUD or a medical backgrounded tended to display a more comprehensive understanding of addiction than others, often citing the complexity of the condition. A common theme emerged around the understanding that many people become unintentionally addicted to drugs, but it is an individual’s initial choice to start using. Some participants explained the far-reaching effect of addiction on their communities, including the impact on family members and friends.

“And now, we see it everywhere. It doesn’t matter, like you said, it does not discriminate. We see it from million-dollar families, million-dollar homes…to someone living out of a car. It doesn’t matter, it’s everywhere.”—LB-01-01

#### Perspectives on linkages to care

Barriers to accessing appropriate care and treatment for addiction were widely acknowledged, from lack of health insurance, to limited facilities, to arbitrary rules preventing people from accessing needed services. One officer explained how a lack of photo identification nearly resulted in a person being turned away from treatment.

“Now [the treatment facility in our county] will only accept people into their program who have multiple forms of ID…birth certificate and something with a photo on it…so I made the kid an ID card at the station, and told the place I’d vouch for him, we knew the kid.”—SG-03-01.

Most participants expressed great frustration with the “revolving doors” on hospitals, some reporting that the person who overdosed had signed themselves out of the hospital before the participant had finished their paperwork. Many participants reported feeling a sense of helplessness when working with PWUD, stemming from a lack of adequate treatment and support options in many regions of the state. Some participants explained new approaches their agencies are taking to divert PWUD away from the criminal justice system and into treatment.

“Instead of rushing out and making arrests on first-time dealings, even with people found to have narcotics on their person, we’re setting up a program that may potentially get them directed to better help, instead of making arrests right from the get-go”-WA-01-01.

However, some participants considered arrest a logical option to “force” an individual using drugs to get the help they need. Drug court was considered by some to be a viable option for getting PWUD linked to critical services. Other participants highlighted concerns around a lack of punishment for those who overdose who are “enabled” by the ready availability of naloxone.

“When we're giving everybody in the world Narcan or naloxone, however they refer to it, the issue becomes, we're now enabling people…because they're saying, hey, you know what, you have a Narcan kit, so if I do overdose you're gonna save my life.” SG-03-01

#### First responder stressors

Generally, participants did not consider responding to the scene of an overdose to be any more of a contributor to work-related stress or trauma than any other calls. Most explained that such stressors were common in their line of work and developing “thicker skin” was a necessity in order to function effectively at the job. Varying levels of support for processing potentially traumatic events at respective agencies were reported. A more personally traumatic event (e.g., the death of a fellow officer) usually results in an immediate response from an agency, with mental health resources made readily available. The day-to-day stressors are not viewed through such a lens; some participants reported feeling little support in this respect, while others were confident that superiors would help them to find the resources if the need arises. A common theme around officer camaraderie did emerge, with many describing in detail the importance of their relationships with other members of the law enforcement community and the need for regular debriefings. Some officers described seeking out others at their agency to debrief, while others went to family members who also worked in law enforcement:

“…really [I process by] just talking about it [the work] with the guys. And my brother's on the job…and he was a paramedic before. So him and I, we talk about everything.”– CD-07-02

## Discussion

Law enforcement officers in NYS are emerging as an integral part of the continuum of care for PWUD based upon their status as first responders and their ability and willingness to administer naloxone. Our findings are capturing a time of transition in which traditional approaches to law enforcement appears to be shifting toward prioritizing prevention for those experiencing drug overdoses. These findings, which offer evidence that law enforcement officers are effective as first responders to suspected opioid overdose incidents, are demonstrative of such transitions. Similarly, attitudes around drug use appear to be evolving. Many officers described factors that have shaped their perspectives over time, seeing drug use as a health problem rather than an immoral or criminal act.

Policing has been referred to as “the public service tasked with response to a range of emergencies involving drug use, homelessness and mental illness…the result of a combination of factors including limited public investment in health and social services…” [27]. Officers are operating in a space where they are expected to enforce drug-related laws while simultaneously managing myriad responsibilities that extend beyond the scope of traditional policing. Widespread implementation of Crisis Intervention Training (CIT) programs, which were developed to reduce the risk of injury or death during an emergency between individuals with mental illness and law enforcement officers, is a testament to the importance of equipping officers with the skills to respond to an array of complex encounters [28]. Although CIT programs show limited effectiveness in terms of reducing lethality during police encounters with people with mental health and substance use disorders, the program succeeded in increasing officer satisfaction and self-perception in the reduction of use of force [28].

A critical element of this discussion includes the perspective of members of the harm reduction community, who acknowledge that collaboration with law enforcement may be “a practical necessity,” but should not take resources away from communities themselves [29] and can be problematic given law enforcement’s role as “the foot soldiers for the drug war” [30]. Additionally, there is a growing body of evidence that documents how policing practices can result in health risks for PWUD, such as people consuming drugs in risky settings to avoid police encounters [31]. Few law enforcement officers would likely consider themselves harm reductionists, although certain acts such as warning PWUD about bad batches of drugs, seeking out particularly violent drug dealers, and responding to calls for overdose assistance indicates that officers contribute to reducing drug-related harms [32]. As one participant stated, naloxone “is just another tool on my toolbelt”. However, training in naloxone administration without addressing stigma and an understanding of drug use behaviors will not necessarily result in naloxone administration nor linkages to care. In a 2013 survey of the Seattle Police Department, half of the 99 officers that shared comments regarding naloxone administration suggested naloxone should be administered by medical professionals only and that naloxone enables drug use [18].

Although disproportionate stress or trauma as a result of responding to calls for overdose assistance was not reported, it is worth noting that repeated exposure to such stressors may inadvertently change attitudes or contribute to occupational burnout. It is possible that repeated exposure to the effects of the opioid crisis may result in compassion fatigue, highlighting the need for additional training and development of coping strategies [33].

Adoption of naloxone administration by law enforcement officers in New York is a powerful example of the successful integration of a public health intervention into police work, and one that continues to adapt to changing circumstances, such as ramping up leave-behind naloxone initiatives. Despite challenges, law enforcement officers, for the most part, have displayed the willingness and ability to execute duties associated with opioid overdose response efforts. We found general acceptance of naloxone administration among the members of the law enforcement community and acknowledgment of an officer’s ever-changing role as a first responder, themes supported by previous research [34]. However, we also found that frustrations remain common among members of the law enforcement community as appropriate treatment options are often lacking, and comprehensive options to address addiction and support PWUD are not easily accessible [34, 35].

### Limitations

Limitations to the study should be noted, specifically the effects of bias associated with the recruitment process. Officers who were trained to administer naloxone but never reported an administration were excluded from the research design; it is possible that negative perspectives toward PWUD prevented them from administering naloxone in the first place. Given that participants were ultimately able to opt in or out of participation in the interviews, self-selection bias may have been introduced. In addition, supervising officers often recommended participants to take part in the interviews. This may have resulted in selection bias on the part of the supervising officer, choosing those officers who would be amenable to questioning on experiences interacting with PWUD.

## Conclusion

Our study findings indicate that although some ambivalence may exist among law enforcement officers, most officers are not deterred from administering naloxone when called upon to do so. Resistance, where noted, is likely explained in part by the historical criminalization of drug use and the associated stigma. Traditional approaches to policing have sought punishment to combat drug use, rather than compassion and access to needed services. Being called upon to be “compassionate warriors” has pushed law enforcement to operate as both crime fighters and social workers, as explained by both Chopko [36] and Manzella and Papazouglou [37]. For some officers, an element of cognitive dissonance may be associated with these, at times, conflicting roles. This begs the question of whether law enforcement involvement is appropriate and viable as a component of harm reduction strategies.

Past research has focused mainly on officer abilities to be trained and administer naloxone and to a lesser extent on their experiences working with PWUD. This study’s findings can aid in the improved design of future public health/public safety collaborations, specifically programs that aim to increase officer involvement in harm reduction work and build positive relationships with PWUD.

## Data Availability

The raw data used (audio files, transcripts) are available from the corresponding author upon reasonable request.

## Abbreviations

PUWD: People who use drugs
NYS: New York state
EMS: Emergency medical services
GSL: Good Samaritan law
NYCDOHMH: New York City Department of Health and Mental Hygiene
NYSDOH: New York State Department of Health
DCJS: Division of Criminal Justice Service
OASAS: Office of Addiction Service and Supports
AED: Automated external defibrillator
CIT: Crisis intervention training
